# Heard, Valued, Empowered: Utilising a Quality Improvement Framework to Improve Trainee Experience

**DOI:** 10.1192/bjo.2022.269

**Published:** 2022-06-20

**Authors:** Elizabeth Andargachew, Ben Pearson-Stuttard, Louise Mowatt

**Affiliations:** St Johns Hospital, Livingston, United Kingdom

## Abstract

**Aims:**

Feedback from doctors in training (DiT) through the Scottish Training Survey has highlighted poor trainee experience within Psychiatry at St. John's Hospital, Livingston. Research suggests that a healthy, happy and engaged workforce experiences lower levels of burnout and provides higher quality patient care. Our aim was to improve the experience of DiT working within the department and thereby improve patient care.

**Methods:**

We utilised the Wellbeing, Conditions and Rota Evaluation (WeCaRE) framework. This is a user-friendly quality improvement (QI) framework designed to improve trainee experience. As part of WeCaRE, questionnaires and ‘what matters to you’ conversations were undertaken with ten DiT (foundation doctors, GP trainees, and core psychiatry trainees). From the issues raised, trainees were empowered to co-create change ideas and use Plan-Do-Study-Act (PDSA) cycles to address the issues. Finally, the questionnaire was repeated to complete the loop.

This approach created an open, listening environment with clear communication channels from trainees to consultants and management. This allowed us to identify themes for improvement. These included induction, education opportunities, clinical supervision and escalation policies.

In collaboration with trainees, three improvement teams were formed, each of which addressed an issue through a PDSA cycle. These were:

1. Unclear referral pathway to Psychiatry resulting in inefficiency. The team co-created a flowchart identifying how to appropriately refer to Psychiatry, which has reduced the number of inappropriate bleeps.
2. Unclear escalation policies and consultant cover. The trainees worked with the multidisciplinary team to generate a clear escalation pathway.
3. Significant variation in content and documentation of clerking – the data collected helped drive change through the utilisation of an electronic clerking checklist.

Other issues were raised and quickly addressed without requiring a PDSA cycle. Such issues included provision of on-call rooms, parking spaces, improvements to induction, starting a Balint Group for trainees, and changing the mode of administration of Pabrinex.

**Results:**

During the five-month period those who experienced joy in work several times a week or more increased from 0%-86%. Those who always felt a valued member of the team increased from 29%- 86%. Those with overall job satisfaction increased from 0%-75%

**Conclusion:**

DiT experience comprises more than rota compliance. It includes well-being, psychological support, professional development, teamship and more. This project has demonstrated considerable improvement in trainee experience through utilising the WeCaRE framework. This highlights the power of listening to, valuing and empowering trainees, whilst utilising data as a vehicle to drive change.

